# Two-Solvent Method Synthesis of NiO/ZnO Nanoparticles Embedded in Mesoporous SBA-15: Photocatalytic Properties Study

**DOI:** 10.1186/s11671-016-1445-2

**Published:** 2016-04-27

**Authors:** Peng Dai, Tao-tao Yan, Xin-xin Yu, Zhi-man Bai, Ming-zai Wu

**Affiliations:** Key laboratory of Information Materials and Device, School of Physics and Materials Science, Anhui University, Hefei, 230039 People’s Republic of China; Engineering Technology Research Center of Magnetic Materials, Hefei, 230039 People’s Republic of China

**Keywords:** Mesoporous silica, Nanocomposites, Photocatalysis, Two-solvent method

## Abstract

Different loadings of NiO/ZnO nanoparticles embedded in mesoporous silica (SBA-15) were prepared via a two-solvent method with the ordered hexagonal mesoporous structure of SBA-15 kept. X-ray diffraction, transmission electron microscope, X-ray photoelectron spectroscopy, diffusive reflective UV-vis spectroscopy, and N2 adsorption porosimetry were employed to characterize the nanocomposites. The results indicate that the ordered hexagonal mesoporous structure of SBA-15 is kept and the absorption band edges of the nanocomposites shift into the ultraviolet light regime. The photocatalytic activity of our samples for degradation of methylene orange was investigated under UV light irradiation, and the results show that the nanocomposites have higher photodegradation ability toward methylene orange than commercial pure P-25. The photocatalytic activity of the nanocomposites was found to be dependent on both the adsorption ability of the SBA-15 and the photocatalytic activity of NiO-ZnO nanoparticles encapsulated in SBA-15. In addition, there is an optimal loading of NiO-ZnO nanoparticles. Too high or low loading will lower the photodegradation ability of the nanocomposites.

## Background

Over the past two decades, semiconductor metal oxides have become the promising photocatalysts in environmental remediation for their nontoxic nature, low cost, and chemical stability [1–3]. Among these semiconductor metal oxides, zinc oxide (ZnO) has been recognized as an excellent material for photocatalytic processes for its high photosensitivity, low cost, and environmental friendliness [4–6]. However, the commercial application of ZnO is still subject to higher band gap (about 3.37 eV) and faster recombination of photogenerated electron-hole pairs. To solve these problems, great efforts have been made to enhance the photocatalytic performance of ZnO nanostructures. Recently, the preparation of ZnO-based heterojunctions including noble metal, and other selected semiconductor with regulated bands, is reported for the enhancement of photocatalytic performance, which can facilitate the mutual transfer of photogenerated electrons and holes and help to suppress the recombination of electron and hole [7, 8]. Among these heterojunctions, NiO-ZnO heterojunctural nanostructures have received special research interest. NiO, a p-type semiconductor (Eg = 3.5 eV) possesses high hole concentration, high hole mobility, and low lattice mismatch with ZnO, which facilitates the formation of *p*-*n* heterojunction with ZnO [9, 10]. Recently, electrospun and thermal oxidization methods have been reported for the preparation of NiO-ZnO heterojunctions with enhanced photocatalytic activities [11, 12]. However, these methods are subject either to high cost equipment or nanoparticle aggregation, which increases the grain size, reduces the specific surface area, and depresses the photocatalytic activity. Therefore, the exploitation of a new way for the facile preparation of NiO-ZnO heterojunctions is still highly desired.

In this paper, a facile impregnation technique named “two-solvent” method was developed for the preparation of NiO-ZnO nanoparticles encapsulated in mesoporous SBA-15 silica. Hydrophobic alkane solvent (*n*-hexane, *n*-pentane, or cyclohexane) were first used to impregnate SBA-15 [13–15], increasing the number of germinal and hydrogen bonded silanol, which can result in better wettability and facilitate the introduction of aqueous solutions into the pores. When metal precursor aqueous solution is introduced, the droplets of the solution are liable to be pushed into the pore channels of SBA-15 for the nonpolar hydrophobic environment and the hydrophilic nature of SBA-15. Thus, guest species can be confined and homogeneously distributed within the pores of SBA-15, which prevents nanoparticle aggregation. To our best knowledge, there has been no report on the preparation of NiO/ZnO nanoparticles encapsulated in SBA-15.

## Methods

### Synthesis of NiO/ZnO/SBA-15 Nanocomposites

All chemicals in this paper are analytical pure grade and used as received. The mesoporous silica SBA-15 was synthesized based on ref [16]. The NiO/ZnO/SBA-15 nanocomposites were fabricated by the two-solvent impregnation method at 298 K [17]. In a typical synthesis, 1 g of extracted mesoporous silica SBA-15 was added into 30 mL of *n*-hexane (hydrophobic solvent) with vigorous stirring of 2 h. Then, 0.98 mL of the mixture solution (hydrophilic solvent) of Zn(NO_3_)_2_·6H_2_O and Ni(NO_3_)_2_·6H_2_O with the atom ratio of Zn to Ni of 1:1 were introduced to the above mixture dropwise with vigorous stirring until a paste-like product turned up, which was dried for 12 h ambiently. Finally, NiO/ZnO/SBA-15 nanocomposites were obtained by calcining the dried product at 550 °C for 5 h in a muffle oven at a ramping rate of 1 °C/min. The NiO/ZnO/SBA-15 nanocomposites with different loading of NiO/ZnO nanoparticles are labeled as *x* % NiO/ZnO/SBA-15, where *x* shows the weight percentage of NiO and ZnO in the nanocomposites (the atom ratio of Zn to Ni is kept to be 1:1). Moreover, 30 % NiO/SBA-15 and 30 % ZnO/SBA-15 were also prepared for the comparative study of photocatalytic properties via the same procedure.

### Characterization and Measurements

X-ray diffraction (XRD) patterns were collected on an 18 kW advanced X-ray diffractometer equipped with Cu K_α_ radiation (*λ* = 1.54056 Å). Transmission electron microscopy (TEM) patterns were obtained with JEOL JEM-2100, operating at 200 kV. Brunauer-Emmett-Teller (BET) surface and the pore structures of the samples were measured at 77 K by a micromeritics ASAP2010 nitrogen isothermal adsorption instrument. The pore volumes were determined at a *P*/*P*_0_ value of 0.97, the specific surface areas were computed with the BET equation, and the mean pore diameters were calculated with the Barrett-Joyner-Halenda (BJH) method based on the adsorption branch of the N_2_ isotherm curve. X-ray photoelectron spectroscopy (XPS) was performed on a PHI-5702 instrument with Al K_α_ radiation as the excitation source (hν = 1486.7 eV). UV-vis absorption spectra were recorded on a shimadzu 240 UV-vis spectrophotometer.

### Photocatalytic Experimental Details

The photocatalytic degradation experiments for methylene orange (MO) were carried out in a self-prepared reactor. In the degradation procedure, samples were immersed in a 50-mL beaker containing 40 mL of MO aqueous solution (20 mg/L). Before the solution was irradiated by a 350 W Xenon lamp, the vertical distance between the solution level and the horizontal plane of the lamp was fixed at 10 cm. At an interval of 10 min, 3 mL of solution was taken out from the reactor. The absorbance of the solution was determined on a UV-vis absorption photometer (UV-3200S, MAPADA analytic apparatus Ltd. Inc., Shanghai, China) at a 464-nm wavelength.

## Results and Discussion

### Characteristic of NiO/ZnO/SBA-15 Nanocomposites

**Fig. 1:**
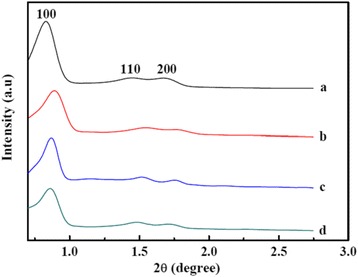
Low-angle XRD patterns of (*a*) SBA-15, (*b*) 20 % NiO/ZnO/SBA-15, (*c*) 30 % NiO/ZnO/SBA-15, and (*d*) 40 % NiO/ZnO/SBA-15

Figure 1 shows the low-angle XRD (LAXRD) patterns of SBA-15 and the nanocomposites with various NiO/ZnO loadings. For pure SBA-15, a strong diffraction peak at 2θ of 0.83° and two weak peaks at 2θ of 1.44° and 1.67° are detected and can be well indexed to (100), (110), and (200) planes of the hexagonal space group (P6 mm), suggesting good long-range order of the as-prepared SBA-15 [16]. Nanocomposites with 20, 30, and 40 % of NiO-ZnO loadings show similar diffraction peaks to SBA-15, indicating that the introduction of NiO/ZnO particles does not destroy the ordered mesoporous structure. The (100) peak at 2θ of 20 % NiO/ZnO/SBA-15, 30 % NiO/ZnO/SBA-15, and 40 % NiO/ZnO/SBA-15 are located at 0.89°, 0.87°, 0.86°, respectively. The *d*_100_ interplanar spacings can be calculated to be 9.9, 10.1, and 10.3 nm for 20 % NiO/ZnO/SBA-15, 30 % NiO/ZnO/SBA-15, and 40 % NiO/ZnO/SBA-15, respectively. Obviously, with the increase of NiO/ZnO loading, the mesoporous structure of the NiO/ZnO/SBA-15 nanocomposites expands [18]. Furthermore, based on the formula ${a}_0=2{d}_{100}/\sqrt{3}$, the unit cell parameter *a*_0_ of samples 20 % NiO/ZnO/SBA-15, 30 % NiO/ZnO/SBA-15, and 40 % NiO/ZnO/SBA-15 are calculated to be 11.4, 11.7, and 11.9, respectively.

**Fig. 2:**
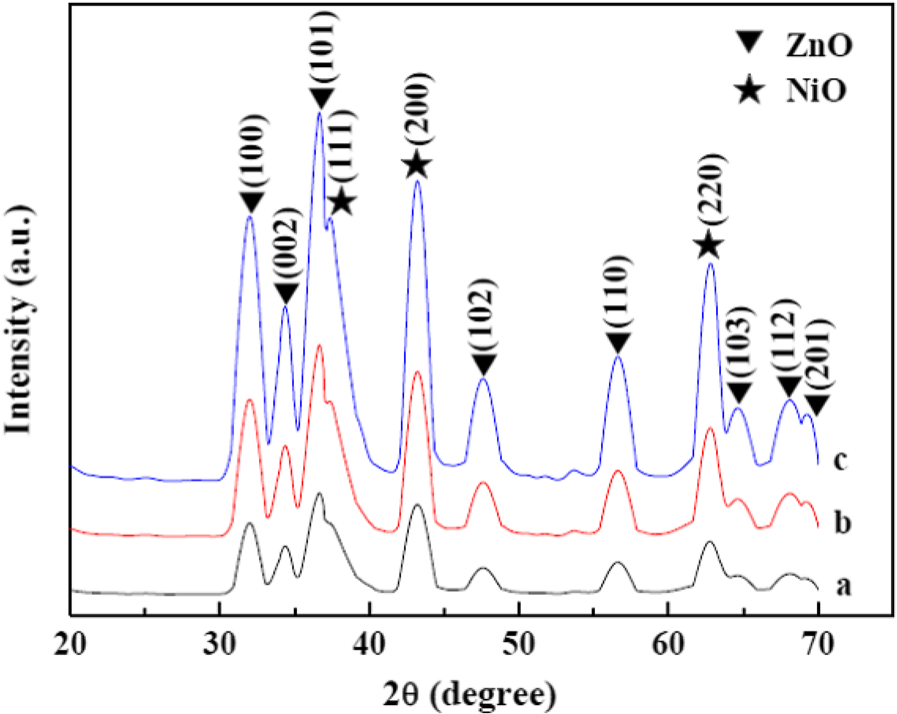
Wide-angle XRD patterns of (*a*) 20 % NiO/ZnO/SBA-15, (*b*) 30 % NiO/ZnO/SBA-15, and (*c*) 40 % NiO/ZnO/SBA-15

Figure 2 shows the wide-angle XRD patterns of samples 20 % NiO/ZnO/SBA-15, 30 % NiO/ZnO/SBA-15, and 40 % NiO/ZnO/SBA-15. Two sets of diffraction peaks can be indexed to cubic NiO (JCPDS card no. 78-0643) and hexagonal ZnO (JCPDS card no. 36-1451), respectively. No peaks of impurity are detected. With the increase of NiO/ZnO loading, peaks become sharper and narrower, suggesting that the crystalline of NiO/ZnO nanoparticles is improved. Based on the Scherrer equation using (200) diffraction peak of NiO, the crystalline sizes of NiO for these three samples are estimated to be 5.0, 5.4, and 5.7 nm, respectively. While for ZnO nanoparticles, the estimated crystalline sizes are 5.2, 5.7, and 6.1 nm for samples of 20 % NiO/ZnO/SBA-15, 30 % NiO/ZnO/SBA-15, and 40 % NiO/ZnO/SBA-15, respectively, using the (100) peak.

**Fig. 3:**
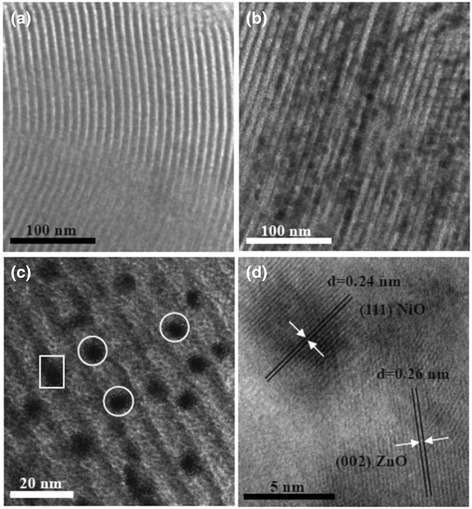
TEM images of SBA-15 and 30 % NiO/ZnO/SBA-15. **a** SBA-15. **b** lower-magnification TEM image of 30 % NiO/ZnO/SBA-15. **c** Higher-magnification TEM image of 30 % NiO/ZnO/SBA-15. **d** HRTEM of 30 % NiO/ZnO/SBA-15

Figure 3 shows TEM images of pure SBA-15 and 30 % NiO/ZnO/SBA-15. For SBA-15, one-dimensional ordered channels are clearly observed, as shown in Fig. 3a. For 30 % NiO/ZnO/SBA-15 sample, two-dimensional hexagonal structure of the silica template is still preserved, after the loading and crystallization of NiO/ZnO with no detected large oxide particles outside the sample surface. Higher magnification image of sample 30 % NiO/ZnO/SBA-15 is shown in Fig. 3c. Clearly, NiO and ZnO nanoparticles are uniformly dispersed in the mesopores of SBA-15, as shown by the circled areas. The size of 8 nm for NiO/ZnO particles is less than the unit cell parameter *a*_0_ (11.7 nm) of SBA-15. Figure 3d further presents the HRTEM image of rectangular area of Fig. 3c. The interplanar distances of 0.24 and 0.26 nm are in good agreement with the lattice spacing of the (111) planes of the cubic NiO and the (002) planes of the hexagonal ZnO, respectively. Obviously, the ZnO particle and NiO particle combine together and form a heterojunction.

**Fig. 4:**
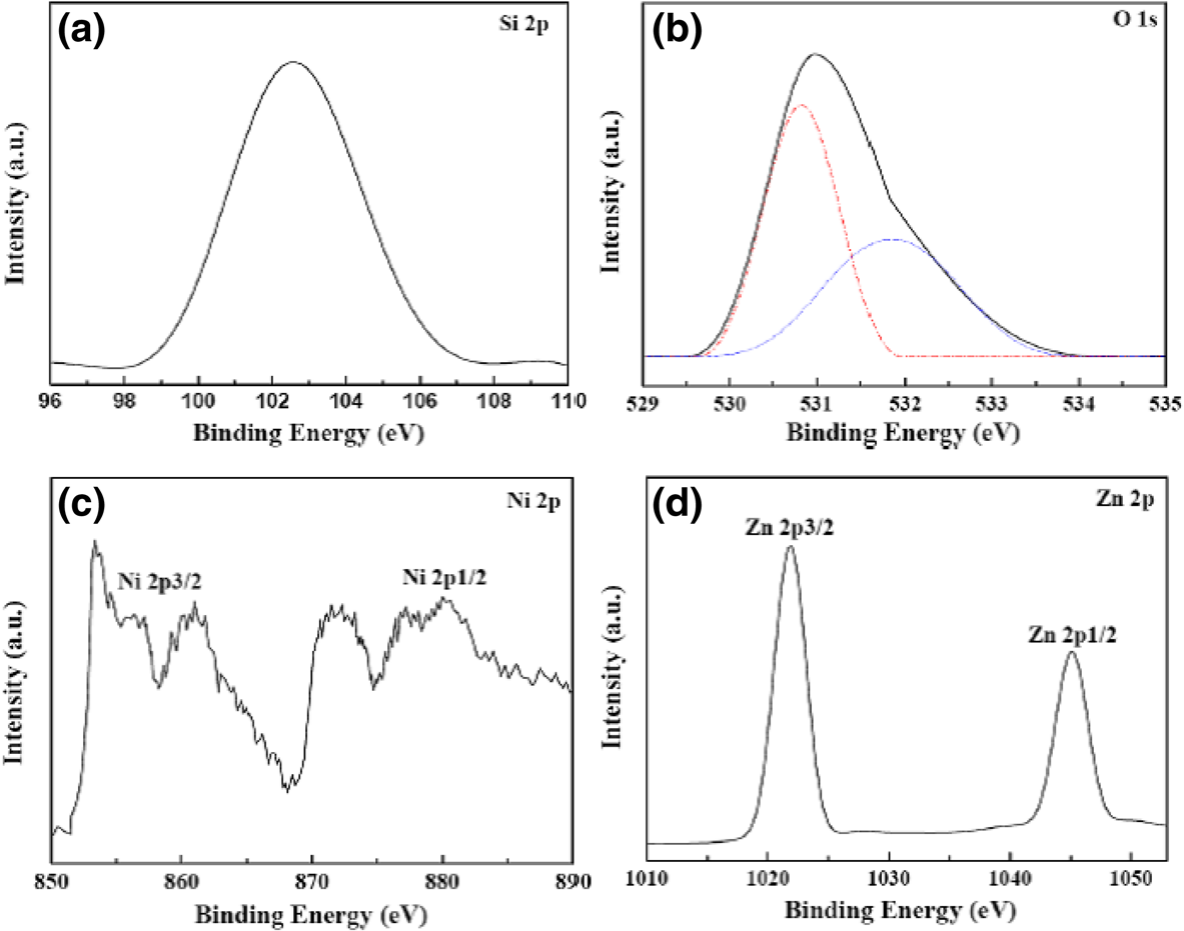
XPS spectra of 30 % NiO/ZnO/SBA-15. **a** Si 2p. **b** O 1 s. **c** Ni 2p. **d** Zn 2p

The chemical composition of sample 30 % NiO/ZnO/SBA-15 was studied by XPS analysis, and the corresponding results are shown in Fig. 4. The peak centered at 102.6 eV is indexed to Si 2p, which is absolutely from silica in SBA-15, as shown in Fig. 4a [19]. Figure 4b shows the O 1 s XPS spectrum, which is asymmetric and abroad, indicating the existence of more than one chemical state for oxygen species. Hence, the region of O 1 s was deconvoluted into two Gaussian peaks centered at 530.8 and 531.9 eV. The lower binding energy at 530.8 eV is attributed to the oxygen in NiO and ZnO [20], while the peak centered at 531.9 eV is due to the adsorbed oxygen in SBA-15 [21]. Figure 4c shows the high-resolution XPS spectrum of the Ni 2p region. The binding energies at around 853.4, 856.0, and 860.1 eV are attributed to the Ni 2p 3/2 peaks, and the 872.2 and 878.7 eV peaks are attributed to the Ni 2p 1/2 peaks, all of which suggests that the oxidation state of Ni is 2^+^ in sample 30 % NiO/ZnO/SBA-15 [22, 23]. In Fig. 4d, the peaks around 1021.9 and 1045.0 eV are attributed to the Zn 2p^3/2^ and Zn 2p^1/2^, indicating the normal oxidation state of Zn^2+^ for our sample [24, 25]. All of the XPS results confirm the successful introduction of NiO and ZnO particles in the channels of SBA-15.

**Fig. 5:**
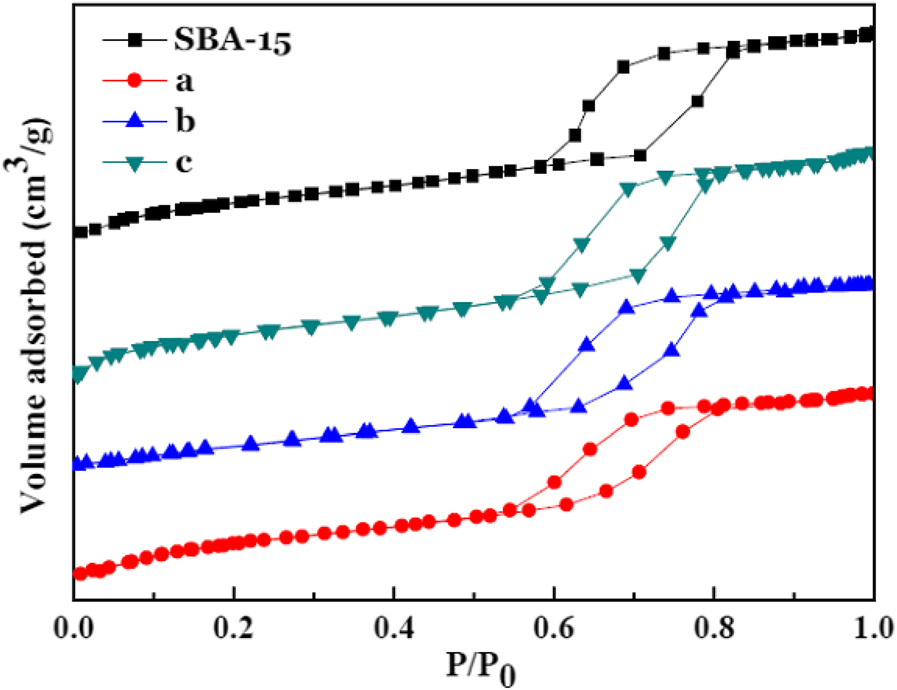
Nitrogen physisorption isotherms of SBA-15 and NiO/ZnO/SBA-15 nanocomposites with different NiO/ZnO loading: (*a*) 20 % NiO/ZnO/SBA-15, (*b*) 30 % NiO/ZnO/SBA-15, and (*c*) 40 % NiO/ZnO/SBA-15

Nitrogen adsorption-desorption isotherms were used to characterize the surface area as well as porosity of samples SBA-15, 20 % NiO/ZnO/SBA-15, 30 % NiO/ZnO/SBA-15, and 40 % NiO/ZnO/SBA-15, and the results are shown in Fig. 5. The isotherms of these four samples show the same type IV isotherm model with a H1-type hysteresis loop, suggesting that all of them are mesoporous materials with two-dimensional hexagonal structures [19]. No obvious changes are detected in the isotherm type, suggesting that ordered mesoporous channels of SBA-15 are well kept after the loading of NiO and ZnO nanoparticles, coincident with the results from the above LAXRD and TEM analysis. Physicochemical parameters of the samples are summarized in Table 1. Clearly, with the increase of NiO and ZnO loading, BET surface area, pore diameter, and pore volume of the samples decrease. In addition, the inflection point of the capillary condensation step on the isotherm shifts to a lower relative pressure with increasing NiO and ZnO loading, suggesting the reduction of mesopore size, which indirectively confirms the existence of NiO and ZnO nanoparticles in the channels of SBA-15.

**Table 1:** Physicochemical parameters derived from nitrogen physisorption and XRD data for different samples (*d*_100_ is the interplanar spacing of the hexagonal structure; *a*_0_ represents the pore-to-pore distance of the hexagonal structure)

Samples	*d*_100_ (nm)	*a*_0_ (nm)	*S*_BET_ (m^2^/g)	Pore diameter (nm)	Pore volume (cm^3^/g)
S0	10.6	12.2	812.5	6.43	0.98
S1	9.9	11.4	698.1	6.19	0.89
S2	10.1	11.7	584.8	6.13	0.80
S4	10.3	11.9	509.3	6.06	0.73

**Fig. 6:**
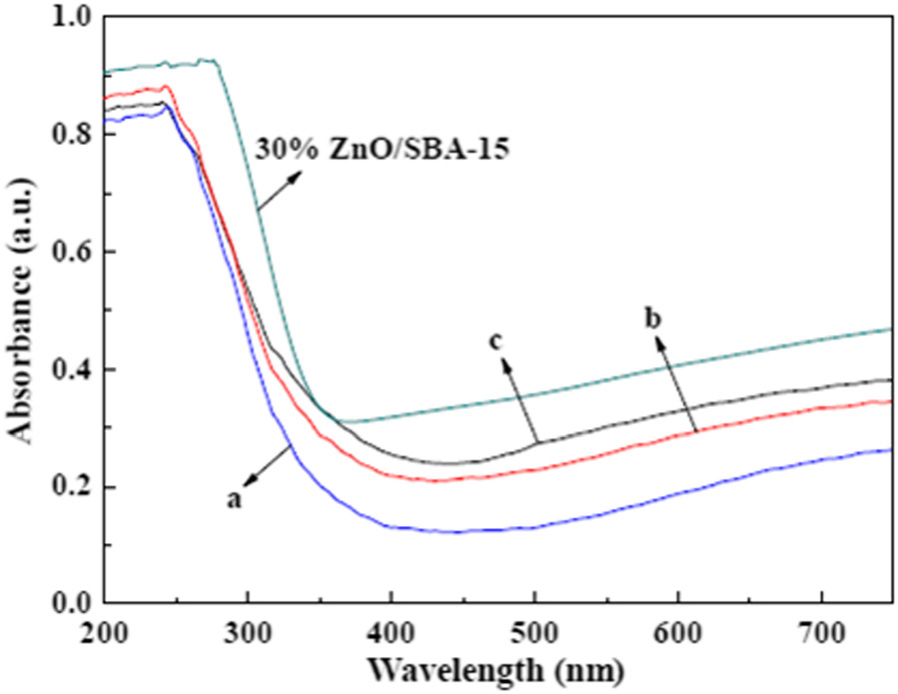
UV-vis absorption spectra of 30 % ZnO/SBA-15 and NiO/ZnO/SBA-15 nanocomposites with different NiO/ZnO loading: (*a*) 20 % NiO/ZnO/SBA-15, (*b*) 30 % NiO/ZnO/SBA-15, and (*c*) 40 % NiO/ZnO/SBA-15

UV-vis spectra of NiO/ZnO/SBA-15 samples with different NiO and ZnO loading are shown in Fig. 6. Clearly, with the increase of NiO and ZnO loading, the absorption band edge shows a red-shift, which can be ascribed to the well-known quantum size effect based on the above XRD analysis and TEM image of crystalline size [26]. Moreover, compared with 30 % ZnO/SBA-15, the absorption band edges of samples extend greatly toward ultraviolet light regime.

### Photocatalytic Measurements on NiO/ZnO/SBA-15 Nanocomposites

**Fig. 7:**
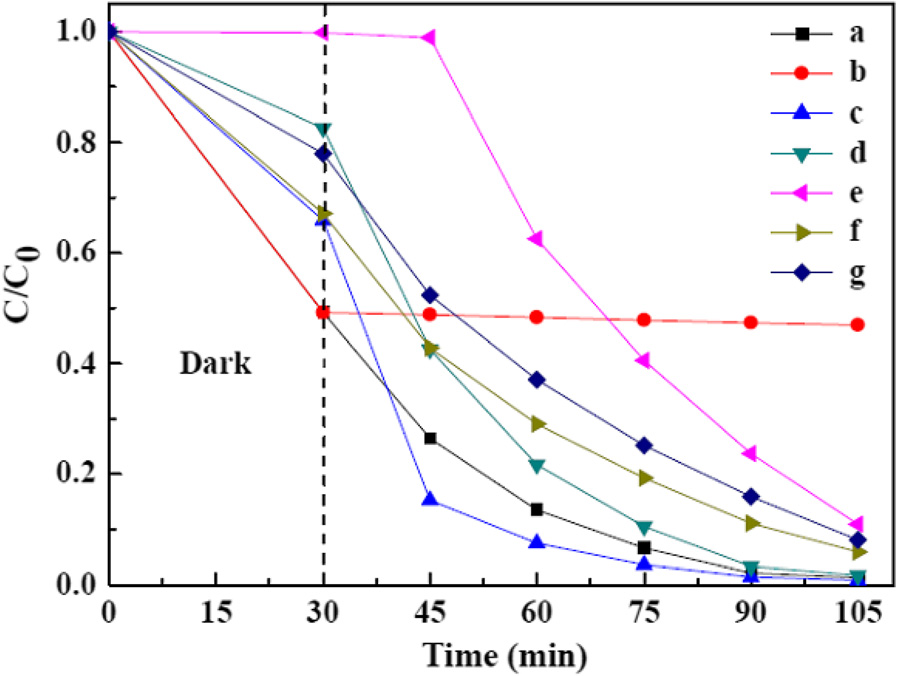
The photocatalytic performance of NiO/ZnO/SBA-15 samples with different NiO/ZnO content: (*a*) 20 % NiO/ZnO/SBA-15, (*b*) 20 % NiO/ZnO/SBA-15 without UV irradiation, (*c*) 30 % NiO/ZnO/SBA-15, (*d*) 40 % NiO/ZnO/SBA-15, (*e*) Degussa P-25, (*f*) 30 wt% NiO/SBA-15, and (*g*) 30 wt% ZnO/SBA-15

To evaluate the photocatalytic activity of the as-prepared NiO/ZnO/SBA-15 nanocomposites, the photodegradation of the well-known organic MO, a typical pollutant in the textile industry, was investigated in water under UV irradiation, and the experimental results are shown in Fig. 7. As an industry standard, Degussa P-25 was chosen for the comparative study. Sample 20 % NiO/ZnO/SBA-15 was used to test the adsorption ability without UV irradiation. As can be seen in Fig. 7, during the initial 30 min, due to the adsorption of MO on the surface of sample 20 % NiO/ZnO/SBA-15, the concentration of MO rapidly decreases and about 50.8 % of MO decolors. After that, the concentration of MO is kept unchanged, suggesting the equilibrium between the adsorption and desorption of MO is achieved. In the absence of UV irradiation, samples 20 % NiO/ZnO/SBA-15, 30 % NiO/ZnO/SBA-15, and 40 % NiO/ZnO/SBA-15 exhibit a much better adsorption capability of MO than that of commercial P-25, which mainly results from their much larger specific surface area and pore volumes. Moreover, 20 % NiO/ZnO/SBA-15 shows higher adsorption capacity of 50.8 % for MO than that of 30 % NiO/ZnO/SBA-15 and 40 % NiO/ZnO/SBA-15 for its highest specific surface area and pore volumes (as shown in Table 1).

**Fig. 8:**
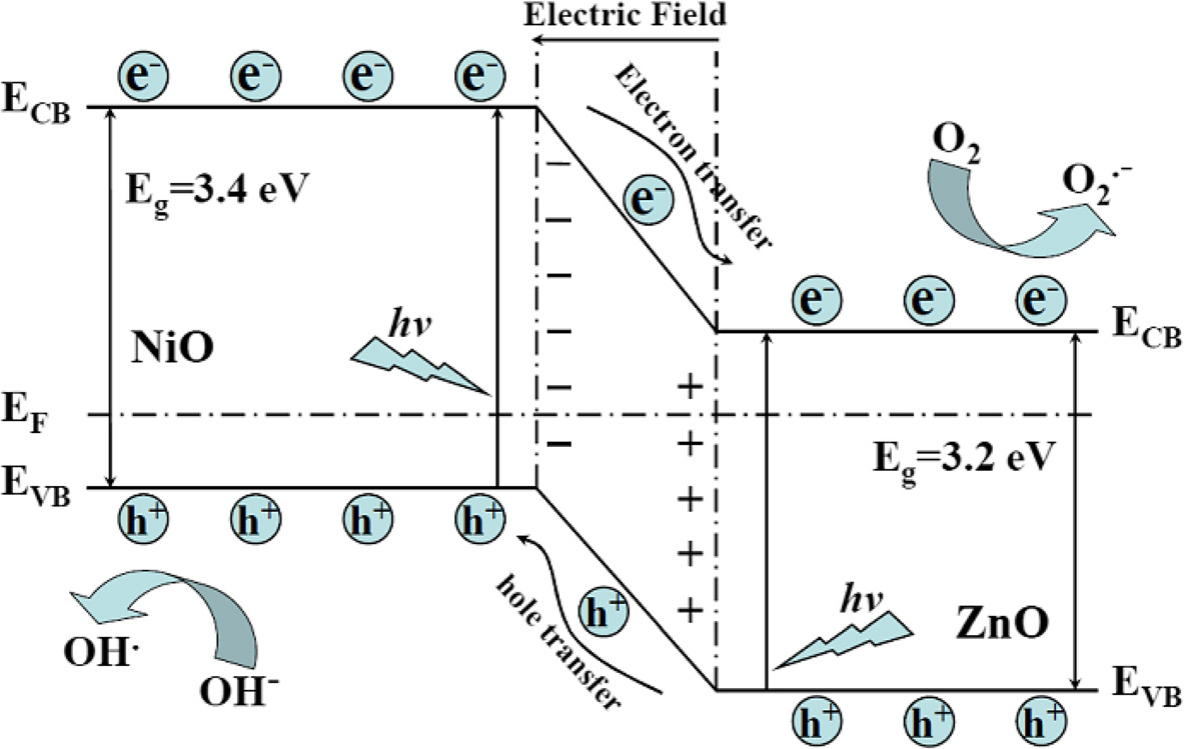
Schematic diagram showing the energy band structure and electron-hole pair separation in NiO/ZnO heterojunction

Based on Langmuir-Hinshelwood kinetics, the rate constants of photodecomposition of MO can be evaluated by the formula [27]:

1 $$ \ln \left({C}_0/C\right)=kt, $$

where *C*_0_ is the initial concentration of the MO solution, *C* is the concentration of the MO solution, *k* is a rate constant, and *t* is the reaction time. After a simple calculation, the values of *k* are 0.0484, 0.0588, 0.0518, 0.0295, 0.0324, and 0.0302 min^−1^ for sample 20 % NiO/ZnO/SBA-15, 30 % NiO/ZnO/SBA-15, 40 % NiO/ZnO/SBA-15, Degussa P-25, 30 % ZnO/SBA-15, and 30 % NiO/SBA-15, respectively. The results show that NiO/ZnO/SBA-15 nanocomposites have better photocatalytic activities than Degussa P-25, and three factors are believed to account for their better photocatalytic activities. Firstly, the large specific surface areas of NiO/ZnO/SBA-15 nanocomposites are beneficial to the adsorption of MO molecules [28]. Secondly, ordered microporous network of NiO/ZnO/SBA-15 nanocomposites can help to promote diffusion and transportation of oxygen species and MO molecules, which greatly enhance photocatalytic activity by facilitating access to reactive sites of NiO/ZnO [29]. Lastly, the NiO/ZnO heterojunction in NiO/ZnO/SBA-15 nanocomposites can improve the separation rate of photogenerated electrons and holes, which reduce the recombination rates and hence improve the photocatalytic activity. Among the three samples of NiO/ZnO/SBA-15 nanocomposites, sample 20 % NiO/ZnO/SBA-15 possesses the lowest *k* value (0.0484 min^−1^), higher than that of 30 % ZnO/SBA-15 (0.0324 min^−1^) and 30 % NiO/SBA-15 (0.0302 min^−1^), which support that the NiO/ZnO heterojunction plays an important role in the improvement of photocatalytic activity. Figure 8 shows the proposed energy band structure diagram of NiO/ZnO heterojunction. The valence band (VB) and conduction band (CB) of ZnO are lower than that of NiO. When the heterojunction is excited by solar light, the photogenerated electron will be transferred from the CB of NiO to the CB of ZnO. Conversely, the photogenerated hole can be transferred from the VB of ZnO to the CB of NiO, indicating that the photogenerated electrons and holes are efficiently separated [30].

Compared with sample 20 % NiO/ZnO/SBA-15 (*k* = 0.0484 min^−1^) and 40 % NiO/ZnO/SBA-15 (*k* = 0.0518 min^−1^), sample 30 % NiO/ZnO/SBA-15 shows the highest photocatalytic activity (*k* = 0.0588 min^−1^), suggesting that there is an optimal loading dosage of NiO/ZnO nanoparticles. In fact, with the decrease of NiO/ZnO loading, the adsorption of NiO/ZnO/SBA-15 nanocomposites becomes increased and the size of NiO/ZnO nanoparticles is decreased. As well known, a higher adsorption is beneficial to the improvement of photoactivity, while smaller nanoparticles in the pore channels of SBA-15 will increase the distance between photoactive sites and the adsorption sites at the surface and enhance the recombination rate of photoinduced electron-hole pairs.

## Conclusions

In summary, NiO/ZnO/SBA-15 nanocomposites with different NiO/ZnO loading were successfully fabricated by using a two-solvent method. The photocatalytic measurement results indicated that the as-obtained NiO/ZnO/SBA-15 nanocomposites possessed higher photocatalytic activity than Degussa P-25 for the degradation of MO dye under UV light irradiation. Higher adsorption performance and enhanced separation efficiency of photogenerated electron-hole pairs are believed to be responsible for the great enhancement of photocatalytic activity. Moreover, there exists an optimal loading of NiO/ZnO for NiO/ZnO/SBA-15 nanocomposites in terms of photocatalytic activity, which is dependent of the adsorption of nanocomposites and NiO/ZnO nanoparticles sizes.
